# Cystic Fibrosis Gene Therapy: Looking Back, Looking Forward

**DOI:** 10.3390/genes9110538

**Published:** 2018-11-07

**Authors:** Ashley L. Cooney, Paul B. McCray, Patrick L. Sinn

**Affiliations:** Department of Pediatrics, Carver College of Medicine, University of Iowa, Iowa City, IA 52242, USA; ashley-peterson@uiowa.edu (A.L.C.); paul-mccray@uiowa.edu (P.B.M.J.)

**Keywords:** history, viral vectors, animal models, clinical trials, adeno-associated virus, adenovirus, lentivirus, retrovirus, non-viral vectors

## Abstract

Cystic fibrosis (CF) is an autosomal recessive disease caused by mutations in the cystic fibrosis transmembrane conductance regulator (*CFTR*) gene that encodes a cAMP-regulated anion channel. Although CF is a multi-organ system disease, most people with CF die of progressive lung disease that begins early in childhood and is characterized by chronic bacterial infection and inflammation. Nearly 90% of people with CF have at least one copy of the ΔF508 mutation, but there are hundreds of *CFTR* mutations that result in a range of disease severities. A *CFTR* gene replacement approach would be efficacious regardless of the disease-causing mutation. After the discovery of the *CFTR* gene in 1989, the in vitro proof-of-concept for gene therapy for CF was quickly established in 1990. In 1993, the first of many gene therapy clinical trials attempted to rescue the CF defect in airway epithelia. Despite the initial enthusiasm, there is still no FDA-approved gene therapy for CF. Here we discuss the history of CF gene therapy, from the discovery of the *CFTR* gene to current state-of-the-art gene delivery vector designs. While implementation of CF gene therapy has proven more challenging than initially envisioned; thanks to continued innovation, it may yet become a reality.

## 1. Introduction: A Brief Summary of Cystic Fibrosis Today

**Figure 1:**
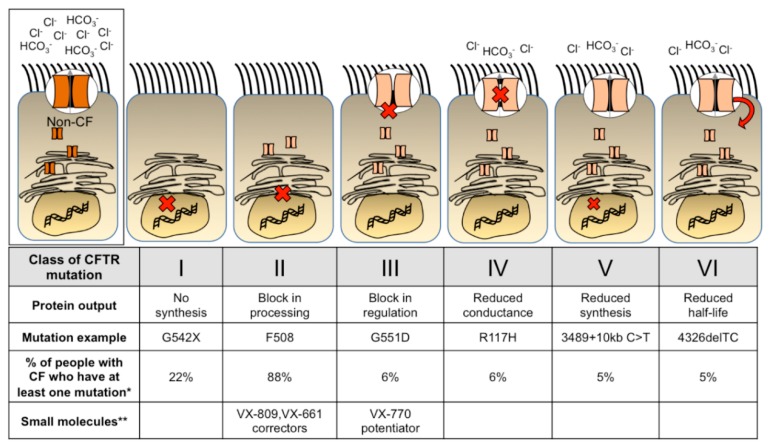
Classes of cystic fibrosis transmembrane conductance regulator (CFTR) mutations. Cystic fibrosis transmembrane conductance regulator mutations are categorized into 6 classes based on the mutation function or protein output [5]. A red “x” or arrow indicates where each CFTR mutant protein is affected. A common mutation example is listed for each class. * People with CF can have more than one mutation; thus, the percentage is representative of the entire population and does not add up to 100. Percentages acquired from the Cystic Fibrosis Foundation (U.S., 2017). ** Potentiators and correctors provide relief to some people with CF in these classes. Additional mutations have been approved for use of CFTR modulators.

Cystic Fibrosis (CF) is a common autosomal recessive genetic disease that affects multiple organ systems. In 1938, Dr. Dorothy Andersen first described the disease as “cystic fibrosis of the pancreas”, which correlated with malnutrition. Since this discovery, aggressive early interventions have been established to improve the quality of life of people with CF, however progressive lung disease remains difficult to manage and is the leading cause of morbidity and mortality. Therapeutic small molecules [1,2,3] provide benefit to a growing percentage of people with CF. Although this is astounding progress, these cystic fibrosis transmembrane conductance regulator (CFTR) modulators are expensive and require lifetime treatment. A one-time treatment administered early in life for people with CF might prevent the onset of lung disease. Figure 1 outlines the six classes of CFTR mutations, examples of each mutation, and the prevalence within the U.S. population of people with CF. Small molecule therapeutics for each class are also labeled. Individuals may have different mutations on each allele and individual mutations may fit into more than one category. Furthermore, new mutation subclasses have been proposed based on the potential corrective therapy potential [4]. As small molecule treatments continue to improve and expand among different mutations, perhaps additional classes or subclasses will be added. Although the development of potentiator and corrector small molecule treatments provide relief for many people with CF, there remains an unmet need for those who have mutations that do not benefit from these treatments. Thus, gene therapy is a mutation agnostic approach and has the potential to repair the phenotypic defect for all people with CF.

CF gene therapy clinical trials pioneered the logistics of endpoint assays and for CF as well as other pulmonary diseases. For example, nasal potential difference (NPD) and forced expiratory volume in 1 s (FEV_1_) were used as clinical outcome measures in CF trials to quantify improvement of lung function (as previously reviewed [6]). The improved quality of life following small molecule treatment is now shedding light on other disease targets, such as inflammation. Lessons learned from these treatments will surely impact future gene therapy clinical trial designs.

**Figure 2:**
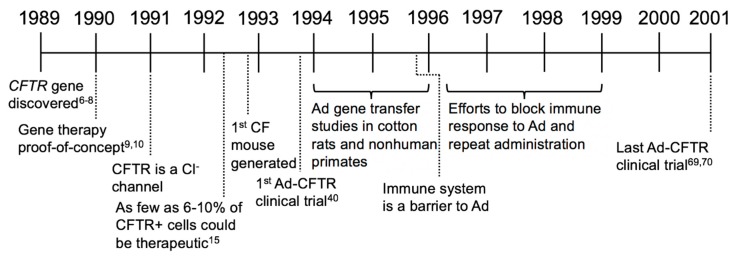
Timelines of CF gene therapy eras: Important milestones impacting the CF field are represented in timelines at the beginning of each era. The timelines are intended to orient the reader to new developments relative to other events and are not comprehensive of all contributions to the field (1989–2001).

In this review, we will focus on the history of CF gene therapy, beginning with the discovery of the *CFTR* gene, continuing with the major milestones that have impacted the field, and looking to the future of CF gene therapy (Figure 2).

## 2. Establishing Benchmarks of Success and Adenovirus-Based Gene Therapy Trials (1989–2001)

In 1989, the gene responsible for CF was identified as *CFTR* [7,8,9]. Sequencing identified multiple *CFTR* mutations, most commonly a three-base deletion that results in the loss of phenylalanine at position 508 (ΔF508) [7,8]. Cloning *CFTR* was a major leap for studying CF and quickly launched the concept of gene-based therapeutics. Within a year of discovering *CFTR*, two groups independently demonstrated the proof-of-concept for gene therapy by expressing full length CFTR complementary DNA (cDNA) in CF cells and restoring anion channel activity. Restoring CFTR activity was proposed as a “cure all” for CF [10,11]. Soon after, several studies using viral and nonviral approaches to deliver *CFTR* and correct the CF defect were advanced, including an adeno-associated virus (AAV)-based vector [12], adenovirus (Ad)-based vector [13], plasmids formulated with cationic liposomes [14], and a retroviral vector [15]. At this time, complementing CFTR in CF patients was considered an achievable, near-term goal.

After ensuring that CFTR complementation restored Cl^−^ current in CF cells, an important next question was to determine the percentage of corrected cells necessary to be therapeutically beneficial. Johnson et al. performed the first studies by mixing CF and MLV-CFTR transduced CF cells in varied ratios in vitro and found that as few as 6–10% of airway cells expressing CFTR achieved non-CF levels of Cl^−^ transport [16]. These studies confirmed that CFTR gene delivery was a potential curative strategy and established a common benchmark of success for gene therapy (i.e., transduction of as few as 6% of airway cells). With relatively good agreement, this and other later studies suggest that expressing CFTR in 5–15% of cells restores Cl^−^ to wild-type levels. Whether this benchmark truly translates into clinical efficacy is an open and complex question; however, as will be discussed, this important question may be addressable with improved gene delivery tools and animal models.

Also during this time, functional CFTR assays and new model systems were being rapidly developed. Experiments were performed on patient-derived immortalized cells such as CF pancreatic cells [17], human bronchial epithelial cells [18], CF bronchial epithelial cells [19], and IB3-1 cells [20]. Established metrics to quantify CFTR expression levels and activity included messenger RNA (mRNA) abundance, the ratio of CFTR protein band C to band B as measured by western blot, in situ hybridization, iodide efflux, patch clamp, and bioelectric properties measured in Ussing chambers [10]. Prior to 1992, no CF animal models existed to test functional gene transfer efficacy in vivo. However, within a short period of time, three groups independently generated CF mice by targeted knockout of endogenous *Cftr* [21,22,23,24,25]. These mice exhibited an increase in steady-state NPD compared to non-CF mice, altered Cl^−^ transport, abnormal mucus accumulation, and disease-related changes in the lung and reproductive tract, but mice did not develop classic CF lung disease. Similar to humans, intestinal obstruction was also reported [26]. Consistent with in vitro experiments, in a CF mouse model, transduction of as few as 5% of cells with a CFTR expressing vector yielded 50% of the non-CF Cl^−^ secretion [27].

At the time these studies were conducted, Ad-based vectors were widely available and production methods were established. Multiple in vivo experiments examined Ad-based lung gene transfer in various models. Repeat doses of Ad-LacZ to cotton rats or nonhuman primates showed that Ad transduced cells within the proximal bronchi and bronchioles including ciliated, secretory, undifferentiated, basal cells [28], and even submucosal glands [29]. The safety of repeated Ad administration was evaluated and studies concluded that Ad delivery was safe, exhibiting little to no immune response upon repeat administration [30,31,32,33]. These and other studies [31,34,35,36,37,38,39,40] suggested that Ad was a promising vector for CF gene therapy.

In 1993, Zabner et al. was the first to publish results from an early CF gene therapy clinical trial [41]. Three CF patients received an Ad vector (serotype 2) carrying *CFTR* [42]. The vector was applied to nasal turbinates with an applicator for 30 min and then removed by suction. One or three days later, one side was biopsied and the contralateral side was used to measure NPD. The authors reported a restored NPD response to a cAMP agonist in the nasal epithelia; however, *CFTR* mRNA and protein were undetectable. In retrospect, airway injury from the delivery method may have facilitated viral transduction. In parallel with the Zabner study, three additional Ad-based clinical trials ensued in efforts to restore CFTR function in CF patients. Crystal et al. performed a phase I dose-escalation study in 4 people with CF and concluded that up to 2 × 10^9^ pfu of Ad2 led to *CFTR* DNA expression in the airway epithelium [43]. One subject that received 2 × 10^9^ pfu in the right lower lobe experienced transient systemic inflammation in the first 24 h, but the symptoms quickly resolved and a 6-month follow up showed no long-term effects [43]. The NPD results in this study were reported as inconclusive but Ad delivery to the nose and lungs appeared to be safe [43]. Knowles et al. used an Ad serotype 5 vector in a larger study of 12 CF participants. Here, Ad-mediated delivery of *CFTR* did not correct the functional defects, perhaps due to inflammatory responses [44,45,46]. Hay et al. delivered Ad5-*CFTR* to nasal epithelia and reported that NPD decreased toward normal compared to the contralateral, untreated nostril [47,48]. In general, these studies suggested that Ad-based vectors could partially correct the Cl^−^ transport defect in CF airway epithelia; however, the effects were transient and inflammatory responses were observed.

Although several studies showed partial CFTR correction in cell and animal models using Ad vectors [49,50,51], the results of clinical trials raised questions about safety and efficacy in humans. Both innate and cellular immune responses presented obstacles to achieving long-term and efficient gene transfer with Ad-based vectors. A nonhuman primate study reported increased alveolar inflammation with high doses of Ad vector [52,53]. Although Ad efficiently delivered genes to the lung, transient expression meant that repeat administration would be required [52,53]. Studies during this time elucidated one mechanism for transgene elimination as mediated by MHC-II presentation of viral antigens which led to activated CD8^+^ T cells in mice [54]. Immune responses also induced neutralizing antibody formation [55]. These serotype-specific neutralizing antibodies against Ad viral capsid antigens were found to prevent readministration of the same Ad serotype [56]. Efforts to facilitate repeat administration included: Blocking IgA antibodies [57], blocking neutralizing antibodies [58], and a nondepleting hCD4 antibody [59], none of which substantially improved repeat Ad administration.

To determine if Ad-vector readministration could achieve long-term CFTR expression in people with CF, clinical trials tested safety and effectiveness of repeat Ad administration. A trial in 1996 featured dose-escalation with 5 repeated doses up to 10^10^ pfu in the nasal epithelium of CF subjects. They concluded that the vector partially corrected the Cl^−^ transport defect but observed significant variability between subjects. Of note, they found less CFTR-correction with each administration, likely due to the immune responses [60,61,62]. Another Ad5 clinical trial by Bellon et al. reported no toxic effects using doses of 10^5^–10^8^ pfu and detectable *CFTR* DNA and mRNA [63,64]. Other clinical trials occurring during this time using Ad5 with additional E3 or E4 deletions reported transient expression after a second administration [65] or low efficiency in the lower respiratory tract with short duration of expression [66]. Overall, repeated doses of Ad were not an effective therapeutic option. 

In 1999, in a clinical trial for ornithine transcarbamylase (OTC) deficiency, an Ad5 vector carrying the OTC gene was delivered to an 18-year-old male subject named Jesse Gelsinger. He received a dose of 3.8 × 10^13^ viral particles via the femoral artery for delivery to the liver [67,68,69]. Four days after administration, he died from multiple organ failure and associated cytokine storm. Although this disease is unrelated to CF, it tragically demonstrated that high systemic doses of Ad can be fatal. In total, 9 CF clinical trials used Ad as the delivery vehicle, the last published in 2001 [70,71].

**Figure 3:**
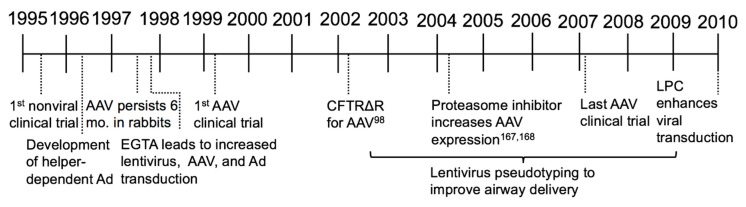
Important milestones for the second era of CF gene therapy (1995–2010).

In the first decade of CF gene therapy the concept of CFTR complementation was firmly established; however, scientists and clinicians learned that delivery of a functional *CFTR* to the airways of humans was more challenging than originally anticipated. CF clinical trials using Ad-based vectors generally supported that gene therapy had potential therapeutic benefit, but improvements in vector design and delivery efficiency were necessary to correct CF lung disease (Figure 3).

## 3. Alternatives to Ad (1995–2008)

The immunogenicity and transient expression of Ad-based vectors were recognized early on. In parallel to many of the aforementioned studies, development of other viral and non-viral vectors was underway. The delivery platforms that received the most attention were (1) AAV-based vectors, (2) non-viral (i.e., plasmid) vectors, and (3) retroviral- (or lentiviral)-based vectors. These vectors were evaluated for their ability to transduce airway cells in vivo and in vitro. Vector modifications and delivery agents were screened with the goal of improving titer, tropism, transduction efficiency, expression, and stability. The barriers to pulmonary gene transfer were examined in greater detail and better measurements for successful gene transfer were invented. In this era, research focused on improving gene delivery, measuring persistent expression, and understanding the host immune response to viral vectors and encoded transgenes.

### 3.1. Adeno-Associated Virus

Similar to Ad, recombinant AAV vectors can transduce terminally differentiated and non-dividing cells [72]. AAV persists episomally and does not integrate in the absence of exogenous Rep protein [73]. In preclinical studies, *CFTR* expression from an AAV serotype 2 vector persisted in rhesus macaques for 3 months [74] and a single delivery to the lung was shown to be safe [75]. In the newborn rabbit, AAV transduced alveolar epithelial cells, tracheobronchial and ciliated lung cells [76,77] and vector DNA was detectable for up to 6 months [78]. Functional CFTR activity was demonstrated in cultured cells by an iodide efflux assay [79]. Although early persistence results were encouraging, AAV expression decreased over time. Loss of expression was attributed to the dilution of transduced cells through lung growth and lack of stem cell transduction [80]. AAV could be readministered and persist for 8 months following transient immunosuppression with an anti-CD40 ligand antibody and a soluble CTLA4-immunoglobulin fusion protein [81]. Thus, a single dose of AAV transduced airways and conferred stable expression for many months.

During this time period, AAV vectors were the forefront of CF gene therapy. Many studies showed their safety and efficacy in repeat dosing to nonhuman primates [82,83] and mice, even in the presence of neutralizing antibodies [84]. Serotype studies in cells cultured from ferrets and pigs showed that AAV1, 2, and 5 transduction patterns closely mirrored human airway epithelia, but mice did not reflect this trend [85,86,87]. AAV6 studies in mice evaluated various promoters, which ultimately led to the generation of a hybrid β-actin promoter that included a β-actin splice donor and β-globin splice acceptor, termed CAG promoter [88]. This promoter substantially increased transgene expression in airway epithelia in vitro [88]. Delivering CFTR with AAV helped uncover features of CFTR biology. For example, AAV-mediated CFTR delivery to mouse airways exposed a correlation between gene transfer and mRNA levels which suggested a regulatory role of low levels of CFTR mRNA as an activator of other chloride channels [89]. Multiple in vitro and in vivo studies suggested AAV could efficiently restore CFTR expression in airway epithelial cells.

In 1999, the first AAV serotype 2 vector clinical trial tested single and two dose treatments, reporting safe dose-dependent gene transfer to the maxillary sinus with little to no cytopathic or host immune response [90]. In the midst of designing and testing new vectors, three additional single dose AAV clinical trials took place [91,92,93]. Results from the dose escalation AAV clinical trials for CF were published in 2004 and 2007 [94,95]. These trials used an AAV carrying the full length CFTR cDNA driven by the promoter activity from the AAV ITR. In total, the AAV clinical trials failed to meet their trial endpoints of detecting *CFTR* DNA from nasal brushings by PCR, a change in NPD, a change in specified metrics as measured by high resolution CT scan, and an improved FEV_1_. In general, AAV delivery was considered safe; however, the resultant levels of CFTR expression were typically below the limit of detection. Despite low CFTR expression levels, the results were encouraging because they demonstrated the safety of the vector. 

A limitation of AAV for CF gene therapy is its small carrying capacity of ~4.6 kb. The AAV inverted terminal repeat (ITR) was shown to possess promoter activity and mediate stable, but low level CFTR expression in vitro [79]. The addition of an internal promoter and polyadenylation signal was necessary but exceeded the carrying capacity [96,97,98]. Deletion analysis showed that removing a portion of the R-domain shortened *CFTR* by nearly 300 base pairs and still retained anion channel function to a similar extent as wild-type CFTR while addressing AAV packaging constraints [99]. Additional studies to shorten other expression cassette components led to the generation of a shortened CMVie (Cytomegalovirus immediate early) promoter and polyA [100]. AAV vectors with these modifications delivered a functional CFTR and corrected the anion channel defect in vitro and the *CFTR* minigene complemented the intestinal defect in CFTR null mice [101]. 

### 3.2. Nonviral Vectors

As an alternative to viral vectors, new focus was directed toward a nonviral strategy for CFTR delivery. Nonviral vector delivery is an enticing approach because there are no size constraints; however, efficient delivery to cells is challenging. This challenge is most commonly addressed by formulating plasmid DNA with a cationic lipid. An important mechanistic study during this time showed that plasmids complexed with lipids traffic to vesicles for unpacking prior to reaching the nucleus [102]. Many cationic lipids tested were reported to be safe plasmid delivery vehicles for gene transfer [103,104,105]. In the first nonviral clinical trial by Caplen et al., a cationic lipid complexed with *CFTR* showed partial NPD correction of cAMP-mediated Cl^−^ transport in the nasal epithelium of six people with CF, that approximated 20% of non-CF levels [106,107,108]. Hyde et al. performed a repeat administration trial with a nonviral complex delivered to the nose and reported no loss of efficacy with repeat administration [109]. Seven subsequent nonviral clinical trials all reported partial, transient expression lasting no longer than 4 weeks [110,111,112,113,114,115,116,117]. After a wave of nonviral clinical trials from 1995 to 2004, there was a lull in activity for nearly a decade to focus on improved delivery and gene expression. In perhaps the most ambitious nonviral clinical trial to date, in 2013 Alton and colleagues nebulized CFTR plasmid formulated with the cationic lipid pGM169/GL67A to the lungs of people with CF and reported a 3.7% increase in FEV_1_ [118,119]. However, despite these encouraging results, a nonviral approach to delivering CFTR was not enough to achieve clear phenotypic correction.

### 3.3. Retroviral and Lentiviral Vectors

In the late 1990s, viral vector delivery methods steadily improved and details of vector/host interactions were elucidated. For example, MuLV-based vectors only integrate into dividing cells. Keratinocyte growth factor (KGF) was found to stimulate proliferation of airway epithelial cells and improve MuLV gene transfer efficacy [120]. Additionally, ethylene glycol-bis(β-aminoethyl ether)-*N*,*N*,*N*′,*N*′-tetraacetic acid (EGTA) treatment or formulation enhanced transepithelial permeability and gene transfer with VSV-G pseudotyped MuLV [121], AAV2 [122] and Ad5 [123]. Other studies using retroviral vectors [124] reported enhanced gene transfer in mice after injury with sulfur dioxide [125]. Retrovirus studies in rabbit tracheal epithelial cells also reported persistent expression following transduction of airway epithelial cells; however, transduction only occurred in wounded trachea [126]. These were the first studies to shed light on how receptor access and cell polarity influenced the transduction efficiency.

Lentiviral vectors were welcomed as a promising therapeutic option because genomic integration could provide long-term expression [127]. Primate and non-primate lentiviral vectors quickly replaced retroviral vectors as they could effectively transduce nondividing airway cells [128]. Specifically, feline immunodeficiency virus (FIV) vectors transduced nondividing airway epithelial cells, persistently expressed transgenes of interest, and corrected the CFTR defect in vitro [129]. Further modifications to lentiviral vectors also improved gene transfer [130]. Although typically pseudotyped with vesicular stomatitis virus (VSVG), its basolateral entry preference hindered efficient airway gene transfer [129,131]. Envelope glycoproteins from respiratory syncytial virus (RSV) [131], Marburg and Ebola virus [132,133], influenza HA-M2 [134], severe acute respiratory syndrome (SARS) spike protein [135], Jaagsiekte sheep retrovirus (JSRV) [136,137], baculovirus GP64 [138], and Sendai F/HN [139,140] were evaluated for apical entry properties. Led by these studies, lentiviruses became a new interest in the field of CF gene therapy.

Many studies confirmed that primate and non-primate lentiviral vectors were promising gene therapy candidates. VSVG-pseudotyped human immunodeficiency virus (HIV)-CFTR with a lysophosphatidylcholine (LPC) pretreatment achieved persistent CFTR expression for at least 12 months in CF-null mice [141,142]. Additionally, the Sendai virus F/HN proteins were used to pseudotype a simian immunodeficiency virus (SIV)-derived lentiviral vector carrying *CFTR* and correct the CF defects in vitro [139,140]. A GP64 pseudotyped FIV was shown to support gene transfer in a pig model [143]. HIV-based lentiviruses also transduced marmoset lungs [144] and achieved long-term correction in CF mice [145].

### 3.4. Addressing Barriers to Gene Transfer

Although much progress was made in understanding viral and nonviral vector delivery methods, several barriers remained. For example, since wild-type adenovirus is a respiratory virus, it was assumed to enter airway epithelial cells from the apical surface. However, the transduction inefficiency in vivo suggested that Ad preferred basolateral entry, perhaps due to receptor-mediated entry [146]. This was supported by the finding that EGTA treatment enhanced Ad gene transfer in mouse airways [147] by disrupting tight junctions [148]. The discovery of the Coxsackievirus and Adenovirus receptor (CAR) in 1997 confirmed that the receptor for Ad2 and Ad5 was indeed localized to the basolateral surface [149]. Studies redirecting CAR to the apical surface of human airway epithelial cells using a glycosylphosphatidylinositol (GPI) linkage increased apical gene transfer [123]. This series of events exemplifies a use of vehicle formulations to overcome physical and immunological barriers to the vector. 

Indeed, many vehicle formulations were evaluated for their ability to improve receptor access or reduce immunostimulation upon entry as a means to increase transduction efficiency. Viral vector formulation with polycations neutralized the negative charge of the vector surface glycoproteins, increasing transduction and transgene expression. Examples included polybrene, protamine, diethylaminoethyl (DEAE)-dextran, poly-L-lysine, and cationic lipids [150,151]. Viscoelastic gel formulations also improved apical gene transfer for more than one vector class [150,151]. Agents such as the natural airway surfactant LPC or the calcium chelator EGTA were employed to transiently disrupt epithelial tight junctions and improved VSVG-HIV entry in vivo [152]. Precipitation of Ad using calcium phosphate enhanced gene transfer by receptor-independent endocytosis [153,154,155,156]. Formulating Ad with dexamethasone to reduce inflammation [157], using polidocanol to disrupt tight junctions [158], and using a u7-peptide to improve apical binding [159] were all methods that enhanced gene transfer. Many of these vehicles and experiments have since become widely used in preclinical studies and played a major role in enhancing receptor access and gene transfer.

Additional efforts to improve transgene delivery and understand CFTR expression were explored through vector design. Selection of promoters and polyA signals with the appropriate strength might improve CFTR expression and increase anion channel expression, potentially achieving therapeutic levels of correction with few cells transduced. The PGK, EF1α, and CMV promoters were compared in context with bovine growth hormone (BgH) or SV40 poly A signals [160,161]. A novel hybrid CMV enhancer/ EF1α (termed hCEF) promoter developed for nonviral clinical trials proved efficacious [162]. Due to the size limits to the AAV packaging capacity, the development of a minimal synthetic promoter F5Tg83 also facilitated improved CFTR expression from an AAV vector [163]. The inclusion of inducible promoters regulated by transgene expression levels and modified CFTR expression cassettes were novel ways to regulate gene expression through vector design [164,165,166]. Selective transgene expression in epithelia was first documented by showing that a cytokeratin 18 promoter drives CFTR expression in airway epithelial cells [167]. 

Several advances in vector design helped AAV gene transfer steadily move forward. Co-delivery of AAV with proteasome inhibitors such as doxorubicin and LLnL increased expression in airway cells by preventing proteasomal degradation of the vector [168,169]. Directed evolution of AAV capsids on human airway epithelial cells led to the development of AAV2.5T capsid with improved apical transduction properties to human airway epithelia [170]. Novel AAV capsid variants derived from AAV1, 6, and 9 were generated by directed evolution to improve CFTR delivery [171]. In a dual reporter gene study in chimpanzee airways, AAV1 was shown to transduce 20-fold higher than AAV5 at 90 days post-delivery [172]. Other studies using AAV showed that incorporating peptide motifs into the AAV capsid improved gene transfer in human airway epithelial cells [173]. 

**Figure 4:**
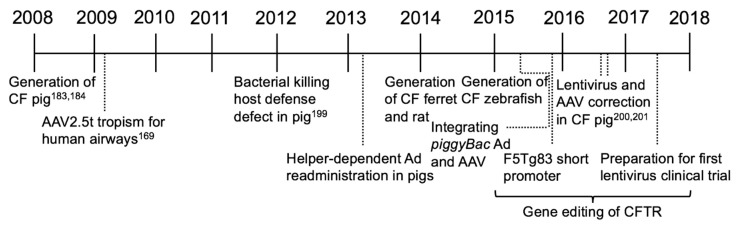
Important milestones for the third era of CF gene therapy (2008–2018).

To circumvent the harmful immunostimulatory properties of Ad, a helper-dependent Ad (HDAd) (also known as delta-rAd or gutted Ad) was developed, expressing no viral proteins [174]. This was a strategy to reduce immune responses to viral antigens in efforts to provide a safer Ad vector option for CF gene therapy [175]. Studies comparing first generation Ad to HDAd focused on understanding the innate immune responses. For example, systemic IL-6 levels were elevated following multiple routes of vector instillation [176], however use of HDAd led to reduced levels of inflammation [177]. Interestingly, both Ad5-CFTR and HDAd5-CFTR decreased lung bacterial infections through restoration of CFTR to the airways in mice [178,179,180,181]. HDAd can be readministered to the mouse lung [182] and reduces the innate immune response [183]. These continued improvements in gene transfer vectors provide an expanded toolbox of delivery options (Figure 4).

## 4. New Models for Preclinical Studies (2008–2018)

In this most recent era of CF gene therapy, it was evident that animal models that developed lung disease would help advance studies of disease pathogenesis and provide a preclinical model to study delivery and efficacy. Because the CF mouse does not develop spontaneous lung disease like people with CF, a model that more closely represents human lung disease would be an advancement. Studies from new models could lead to a better understanding at the basic level of the relationship between loss of CFTR and development of lung disease. 

Several CF animal models are now available. In 2008, the development of a CF pig was reported [184,185]. The pig was chosen because it is a large animal model with many pulmonary, anatomical, physiological, and biochemical similarities to humans. Unlike CF mice, the CF pig recapitulates several features of human lung disease [185,186]. Development of this model has changed the way we think about CF gene therapy. One limitation of the CF pig was the 100% penetrance of meconium ileus that required surgical intervention for long term survival. Creating a “gut corrected” CF pig expressing CFTR in the gastrointestinal tract by the fatty acid binding protein (FABP) promoter alleviated meconium ileus, making longitudinal studies more feasible [187]. 

A CF ferret first reported in 2008 is another large animal model with a lung disease phenotype [188,189]. CFTR-knockout ferrets recapitulated many disease similarities to humans including defective airway Cl^−^ transport, meconium ileus, and the spontaneous development of bacterial lung infections [190]. Studies in non-CF ferrets show that rAAV2/1 and lentiviral vectors facilitate gene transfer in newborn airways, and thus hold promise as vector platforms for CF studies. In addition, a novel parvovirus vector derived from human bocavirus transduces ferret airways [191].

Other newer CF animal models include the rat [192,193], zebrafish [194], and sheep [195]. The CFTR null rat was created in 2014 using zinc finger nucleases (ZFNs) for gene targeting and exhibits an NPD and bioelectric properties similar to humans. Additionally, histological abnormalities in the ileum parallel intestinal complications seen in other animal models. Efforts to characterize a CF rabbit model are ongoing [196]. Novel CF mouse models have been generated to study specific CFTR mutations such as the premature stop codon G542X [197] and gating effects caused by R117H [198]. In addition, a conditional null allele for *Cftr* in mice was generated [199]. Advancements in animal models have provided the essential insight into underlying causes of CF. 

An important advance in understanding CF disease pathogenesis was elucidated using the CF pig by Pezzulo et al. in 2012. Here, they provided evidence that a reduced airway surface liquid pH decreased the bacterial killing ability in newborn CF pigs [200]. From these studies, relevant assays were devised to measure CFTR correction in vivo. These assays were subsequently used to show that delivery of CFTR by FIV [201], AAV [202], and Ad [203] vectors to CF pig airways partially restored anion channel activity, increased the airway surface liquid (ASL) pH, and improved bacterial killing ability. Further understanding of CF host defenses was applied from these studies to define relationships between CFTR expression and Cl^−^ and HCO_3_^−^ transport that have important implications for CF gene therapies [204]. 

### Gene Editing and the Era of Precision Medicine

As a complementary approach to gene addition, the ability to enzymatically modify nucleic acids sequences has advanced a new field of study in the CF research community. Gene editing tools include ZFNs [205], TALENs [206], meganucleases [207], and CRISPR/Cas9 [208]. Zinc finger nucleases were early gene editing tools; however, the ease of CRISPR/Cas9 engineering and guide RNA production allowed broader access of genome modification tools. Gene editing holds the promise of repairing the endogenous *CFTR* gene; restoring CFTR function, for repair of individual mutation, insertion, or partial or full-length cDNA to “safe-site” targeted integration. However, in vivo gene repair faces obstacles that extend beyond those associated with “simple” gene delivery. For each *CFTR* mutation, the efficiency of introducing double stranded breaks with different guide RNAs and the efficiency of homologous recombination with different repair templates will need to be assessed. *CFTR* gene editing by both ZFNs [209,210], TALEN [211], and CRISPR/Cas9 [212] methods have been evaluated in vitro. Importantly, both gene addition and gene repair strategies rely on efficient in vivo gene delivery. Interests include both delivery to respiratory epithelia, and systemic delivery strategies that might correct CF defects in multiple affected organs.

Whether the goal is gene addition or gene repair, for gene therapy to be successful, the vector selected must either integrate into a basal cell or other progenitor cell types or be readministered. While AAV, Ad, and HDAd are generally considered to be non-integrating, development of a hybrid nonviral transposon/viral integrating vector system led to persistent expression in mice for at least a year. Previously, the DNA transposon *piggyBac* has been shown to promote persistent gene transfer in mice [213]. In this system, the *piggyBac*/AAV and *piggyBac*/Ad vector system confers persistent expression in mouse airways only in the presence of the transposase [214]. A recent study in the pig model documents whole lung distribution by a *piggyBac*/Ad vector and phenotypic correction of a CF pig model [203]. Promising work evaluating HDAd suggests that this vector efficiently transduces airway epithelia [215], including submucosal glands [216], and can be readministered with use of an immunosuppressant [217,218]. Perhaps an integrating *piggyBac*/HDAd could provide long-term, efficient correction and be readministered if necessary.

Advances in gene therapy from other genetic disease continue to inform the field. In December 2017, the FDA approved voretigene neparvovec-rzyl (Luxturna) as a gene therapy treatment for individuals with inherited retinal diseases such as retinitis pigmentosa and Leber’s congential amaurosis. For this treatment, an AAV2 vector carrying RPE65 is delivered by injection into the subretinal space [219]. Individuals who received this treatment exhibited increased full-field light sensitivity and improved fMRI results [220]. Exciting advancements in CAR-T therapy for cancer treatments include tisagenlecleucel (Kymriah) [221] and axicabtagene ciloleucel (Yescarta) [222]. For both, anti-CD19 chimeric antigen receptors are delivered by lentiviral vectors to autologous T cells, with some patients achieving complete remission. Gene therapies approved for use in Europe include Alofisel, Spherox, Zalmoxis, Stimvelis, Imlygic, and Holoclar (European Medicines Agency). The steadily growing list of clinically approved gene therapy therapeutics suggests that we may be entering a new era where gene therapy is a commonplace option. 

Currently, there are exciting new developments in CF gene therapy. Nanoparticle delivery chemistries with enhanced tropism are emerging [223] and CF clinical trials using lentiviral vectors are currently being pursued [224]. The major breakthroughs in gene therapy for other genetic diseases is fueling new interest in CF. The field of gene therapy has learned valuable lessons from the many viral and nonviral clinical trials to date and has developed new vectors that can efficiently and persistently correct the CFTR anion channel defect. New animal models that recapitulate features of human lung disease allow preclinical studies to refine delivery protocols and measure novel metrics of phenotypic correction. The early surge in enthusiasm to develop a gene therapy treatment for CF fell to the realization that delivering CFTR to the lung was more complex than expected. Improvements in vector design and delivery methods, understanding lung biology and disease development, and the ability to use new animal models are all reenergizing the gene therapy field and adding to the momentum that we hope will lead to the generation of an effective therapeutic reagent for CF.
